# Trends, Differentials, and Social Determinants of Maternal Health Care Services Utilization in Rural India: An Analysis from Pooled Data

**DOI:** 10.1089/whr.2019.0022

**Published:** 2020-06-23

**Authors:** Arvind Kumar Yadav, Bhavna Sahni, Pabitra Kumar Jena, Dinesh Kumar, Kiran Bala

**Affiliations:** ^1^ Department of Economics, Shri Mata Vaishno Devi University, Katra, J&K, India.; ^2^ Department of Community Medicine, Government Medical College, Jammu, India.

**Keywords:** full antenatal care, India, maternal health care, postnatal care, skilled birth attendance, village level factors

## Abstract

**Background:**

Millennium development goal 5 aimed at reduction of maternal deaths by three-quarters from 1990 to 2015: a target India commendably achieved, but this milestone remains overshadowed by inequalities in utilization of health services that are driven by determinants both at community and at individual level.

**Materials and Methods:**

We studied the utilization trends using descriptive statistics and analyzed the relative contribution of various socioeconomic predictors on the use of maternal health care services in rural India using binary logistic regression analysis on pooled data from three rounds of National Family Health Survey. Outcome variables included four or more antenatal care visits, skilled birth attendance, and postnatal care.

**Results:**

Although utilization of maternal health care services showed an upward trend from 1998–1999 to 2015–2016, factors such as illiteracy, female age ≥40 years, having five and more children, belonging to scheduled tribes, rural residence, and not possessing a health card were associated with significantly low utilization of maternal health care services. However, partner's education, good economic status, women's autonomy, and infrastructure at village level were associated with better odds of availing these services.

**Conclusions:**

The study generates evidence on the role of various socioeconomic determinants in maternal health care utilization and identifies gaps that must be strategically addressed to reach sustainable developmental goal maternal mortality target of 70 deaths per 100,000 live births by 2030. It reemphasizes the need for ensuring convergence among different stakeholders while structuring maternal health policies so that health reforms can be accomplished effectively at all levels of health care.

## Introduction

Maternal mortality remains a major public health concern worldwide, especially in developing countries, hence its mention in the top 5 millennium development goals (MDGs) and in sustainable developmental goals (SDGs). Maternal mortality ratio (MMR) reduced from 556 per 100,000 live births in 1990 to 130 per 100,000 live births in 2016^1^, but despite the considerable improvement in maternal mortality, India still continues to contribute one-fifth of the global burden of absolute maternal deaths.^2^ It has been documented that MMR is higher for women living in rural areas and in poorer communities^3^ coupled with huge disparities across states and social strata. Nevertheless, most maternal deaths are preventable, as the health care solutions to prevent, diagnose, and manage complications are well recognized.^4^ Basic maternal services such as antenatal care (ANC), skilled birth attendance, and postnatal care (PNC) are crucial for reducing and managing pregnancy complications, and for reducing the burden of these preventable deaths.^5,6^ Furthermore, the National Health Policy, 2017, envisages reducing MMR to 100 by 2020 and sustaining antenatal coverage at 90% and skilled attendance of birth >90% by 2025 (Ref.^7^). However, in India, currently only 51% of mothers receive four antenatal checkups, 79% of births occur in health centers, 81% of deliveries are assisted by health professionals, and 65% of women receive PNC.^8^ These gaps in the coverage of essential maternal health care services are a barrier that impedes India's progress toward attaining MMR figures comparable with those of other major economies in the world. Hence there is a pressing need to identify and address the grass root determinants of maternal health care utilization to steer the health services toward achieving the ambitious SDG target of reducing the global MMR to <70 per 100 000 births, with no country having a maternal mortality rate of more than twice the global average by 2030 (Ref.^4^). Also, maternal death is a relatively rare event and its trends over time are difficult to measure, but data on skilled birth attendance are readily available and are often used as a proxy indicator to track progress toward MDG 5.^9^

Several studies in the past two decades have identified three key factors to reduce maternal deaths and improve newborn health. These factors include family planning, deliveries assisted by health professionals, and the availability and accessibility of emergency obstetric care for all the mothers with life-threatening complications. However, World Health Organization (WHO) recommends that three elements of maternal health namely four or more ANC visits, delivery assisted by skilled birth attendants (SBAs), and three postnatal checkups are pivotal in any safe motherhood program in a developing country like India.

The existing evidence suggests a strong concordance among different socioeconomic and demographic factors and the utilization of maternal health care services. The public health and sociological literature refers to the theoretical background about human behavior within social and economic contexts.^10,11^ The theoretical concept given by Giddens^10^ points out that human behavior must be studied at an individual level, including their social, cultural, political, and physical environment. The sociological model^11^ explains individual biological and psychological factors, in relation to regional, technological, and sociocultural factors, which together influence health and health-related behaviors. It is well documented that individual factors such as female literacy have a strong association with maternal health care utilization.^12–15^ Maternal education may also enhance maternal health through women's autonomy.^16^ A decent socioeconomic status empowers couples to use family welfare methods that by default reduce maternal mortality. The village level factors such as public health infrastructure, availability of physical services, and economic development have a strong effect on maternal health care and health outcomes.^17–19^ Studies have found that improved public sector facilities augment utilization of maternal health care services, especially among marginalized women in rural areas.^20^ Infrastructure development and investment in equipment also increase the use of maternal health care.^21,22^

Various governmental interventions over the past two decades have led to the abatement of maternal mortality in India. This reduction, however, remains moderate and irregular, both across and within different countries.^23–25^ A centrally sponsored scheme, the National Rural Health Mission (NRHM), was launched in 2005 to provide effective health care to rural population with special focus on 18 states with poor public health infrastructure. The NRHM attempts to address health challenges with emphasis on improving determinants of health, such as safe drinking water, sanitation, and hygiene and nutrition. The key goal of NRHM was to reduce maternal and child mortality and improve the availability, accessibility, affordability, and quality of effective health care services in rural areas,^26^ particularly among the poor and deprived populations. In addition, Janani Suraksha Yojana was launched under the broad umbrella of NHRM, offering conditional cash benefits on institutional delivery among women in below poverty line families.

There is a dearth of empirical research on the utilization of maternal health care services in rural India. It is necessary to know whether the use of maternal health care services in rural areas is enough. Few pertinent questions are what are the trends and patterns of maternal health services, what factors contribute to the use of these services, and do village level factors contribute significantly? This study tries to answer these questions and suggest appropriate policy measures by using data generated through National Family Health Survey (NFHS). Thus, the major contribution of this study is (1) to study the trends of maternal health care services utilization in India, (2) to find out the differentials and status in use of maternal services with regard to socioeconomic and demographic indicators, (3) and to assess and analyze the effects of individual and village level factors on the utilization of maternal health care services in India.

## Data and Methodology

This study used pooled data from three rounds of NFHS carried out during 1998–1999, 2005–2006, and 2015–2016. NFHS is a nationally representative survey and covers >99% of Indian population. NFHS provides reliable estimates of pregnancy complications, frequency of ANC, place of delivery, PNC, and maternal health care services assisted by skilled health professionals and other predictors at national and state level.

### Outcome variables

The study measures three outcome variables, namely, four or more ANC visits, skilled birth attendance, and PNC. The use of these three indicators of maternal health care utilization is based on the key guidelines developed by WHO^27^ and Ministry of Health and Family Welfare, Government of India.^28^ An SBA is defined as “qualified health professional like doctors, midwife, or nurse who has been educated and trained to proficiency in the skills needed to manage normal (uncomplicated) pregnancies, childbirth and the immediate postnatal period, and in the identification, management and referral of complications in women and newborns.” Delivery assisted by skilled health professionals [doctor/nurse/lady health visitor/auxiliary nurse midwife (ANM)/other health professionals] is considered as safe delivery (NFHS, 2015–2016). This study measures PNC received within 2 days after delivery, but some studies have documented postnatal visit at 6 weeks.^29,30^

### Defining key explanatory variables

The study includes socioeconomic and demographic predictors such as women's education (no education, primary, secondary, and higher), women's age (15–19, 20–29, 30–39, and 40–49 years), birth order (1, 2–4, 5 and more), health card (yes vs. no), partner's education (no education, primary, secondary, and higher), religion (Hindu, Muslim, and Others), and caste (scheduled castes, scheduled tribes, other backward class, and upper caste Hindu). Women's autonomy was estimated by using three parameters, namely, women's mobility (permission to go to health center), access to economic resources (has a bank account), and mass media exposure (watching a television once in a week and reading newspaper once in a week). Village level factors included availability of health infrastructure at rural areas, drainage facility, village electrification, availability of clean water, transport facility, and standard of living of villagers (standard of living of the villagers is based on wealth index). This study used village level factors as a proxy.

### Statistical approach

This study used weighted sampling to generalize the results at the national level. Pooled data were used for regression analysis, since pooled regression gives more robust results. The study used pooled data of only three rounds of NFHS as the outcome variables such as ANC visits, SBA, and PNC were similar. The first round of NFHS conducted during 1992–1992 did not cover PNC, hence this study did not include data from the first round of NFHS (1992–1993). Binary logistic regression model was used to observe which factors best predict and explain the utilization of ANC, skilled birth attendance, and PNC.

## Results

### Trends of maternal health care services utilization

**FIG. 1.**
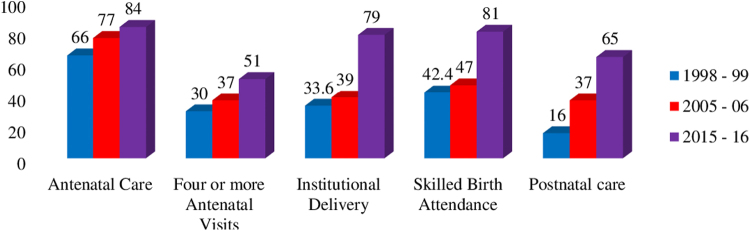
Trends of maternal health care services utilization in India. NFHS, National Family Health Survey.

The data in Figure 1 show the trends in utilization of maternal health care services. There was a steady rise in four or more ANC visits from 30% in 1998–1999 to 51% in 2015–2016. Institutional deliveries went up from 33.6% in 1998–1999 to 79% in 2015–2016. During the same period, skilled birth attendance approximately doubled and PNC quadrupled from 16% in 1998–1999 to 65% in 2015–2016.

### Utilization of maternal health care services (in percentage) by socioeconomic characteristics

Table 1 shows how service utilization and delivery vary with various socioeconomic and demographic indicators. Findings reveal that women with no education, aged ≥40 years, having five and more children, women belonging to scheduled tribes, and those residing in rural areas reported low utilization of maternal health care services. It was observed that four or more antenatal checkups were one corollary of higher education among women. It is evident that 73%, 88.2%, and 72.7% of women with higher education had made four or more ANC visits as compared with 12.4%, 20.8%, and 29.23% of those with no education in 1998–1999, 2005–2006, and 2015–2016, respectively. In total, 22.5%, 34%, and 68.5% of younger women (aged 15–19 years) reported receiving four or more antenatal checkups as compared with 6.1%, 25.2%, and 63.5% of the older women aged 40–49 years in 1998–1999, 2005–2006, and 2015–2016, respectively. A higher percentage of women with birth order 1 (28.9%, 45.2%, and 78.4%) had made four or more ANC visits as compared with women with birth order of ≥5 (27.7%, 44%, and 42.3%) in 1998–1999, 2005–2006, and 2015–2016, respectively. In NFHS 4 data, there existed a disparity in the expected trend among women of different income groups with regard to four or more antenatal checkups. Most of the women from highest income groups had received adequate ANC namely 66.3% in 1998–1999, 84.7% in 2005–2006, and 75.9% in 2015–2016 as compared with women belonging to poorest strata (24.5% in 1998–1999, 48.7% in 2005–2006, and 28.8% in 2015–2016, respectively). A higher percentage of women from urban areas made four or more ANC visits as compared with rural areas from 1998–1999 to 2015–2016.

**Table 1.** Utilization of Maternal Health Care Services (in percentage) by Socioeconomic Characteristics

	4+ ANC			SBA			PNC		
	1998–1999	2005–2006	2015–2016	1998–1999	2005–2006	2015–2016	1998–1999	2005–2006	2015–2016
Women's education									
Illiterate	12.4	20.8	29.23	15.3	26.1	66	24.9	24.0	51.0
Primary	31.7	38.4	43.5	35.7	45	74.1	27.3	39.8	59.4
Secondary	49.3	63.0	59.6	53.8	80.3	92.3	30.9	69.3	73.5
Higher	73.0	88.2	72.7	70.9	91	94.9	38.7	82.8	77.6
Women's age (years)									
15–19	22.5	34.0	68.5	25.9	47.2	83.7	33.3	39.0	65.5
20–29	31.4	47.8	53.5	34.3	46.4	81	29.9	44.2	67.4
30–39	21.1	45.1	51.5	26.0	51.5	77	32.0	36.8	57.3
40–49	6.1	25.2	63.5	3.0	73.4	66	38.6	26.0	54.6
Parity									
1	28.9	45.2	78.4	31.5	65.2	89	19.9	56.7	72.1
2–4	29.9	45.8	54.5	32.6	47.7	81	18.0	46.3	65.2
5+	27.7	44.0	42.3	32.8	37.3	65	14.3	26.3	51.3
Income									
Poorest	21.5	48.7	28.8	29.8	19.4	64	15.6	19.3	47.9
Poorer	26.9	59.3	37.8	30.9	31.8	78	16.0	27.7	59.9
Middle	30.2	70.7	47	30.8	49	87	16.5	42.9	69.4
Richer	44.1	75.6	59.8	33.0	67.2	92	18.0	56.8	75.0
Richest	66.3	84.7	75.9	34.9	88.8	96	20.5	79.3	79.5
Religion									
Hindu	37.8	72.5	53	31.30	47.5	83	16	42.9	65.8
Muslim	31.7	68.3	51	31.69	38.8	73.6	15.7	35.9	58.9
Others	26.8	40.2	52	37.40	61.88	86	21	53.0	74.6
Caste									
Scheduled caste	23	67.1	42.9	27.16	40.6	80.7	17	37.1	64.4
Scheduled tribes	21.1	58.6	54.8	22.29	25.4	71.5	14	31.4	59.0
Other backward class	31.3	67.8	57.6	35.55	46.7	82	16	40.2	64.9
Upper caste	33.9	73.2	56.2	37.21	57.8	85.3	18	52.2	69.1
Place of residence									
Rural	20.8	65.8	37.5	22.7	37.5	78	16.1	33.9	61.7
Urban	52.9	72.2	57.8	60.1	73.5	90	19.6	65.7	73.1

*Source:* Data based on three rounds of NFHS.

ANC, antenatal care; NFHS, National Family Health Survey; PNC, postnatal care; SBA, skilled birth attendant.

It was noted that 70.9%, 91%, and 94.9% of women with higher education reported skilled birth attendance as compared with 15.3%, 26.1%, and 66% in 1998–1999, 2005–2006, and 2015–2016, respectively. In total, 25.9%, 47.2%, and 83.7% of younger women (aged 15–19 years) delivered their child in the presence of an SBA as compared with 3%, 73.4%, and 66% (aged 40–49 years) in 1998–1999, 2005–2006, and 2015–2016, respectively. There was wide variation among the women who had delivered their child in the presence of an SBA by income quintile. As income increased from poorest to richest, the deliveries assisted by SBAs also followed an upward trend. In total, 34.9%, 88.8%, and 96% of the richest women reported skilled birth attendance as compared with (29.8%, 19.4%, and 64%) poorest women in 1998–1999, 2005–2006, and 2015–2016, respectively. Slight differences in skilled birth attendance were noticed in relation to religion and caste. However, a huge rural–urban variation was observed in terms skilled birth attendance. In total, 60.1%, 73.5%, and 90% women belonging to urban areas had delivered their child in the presence of SBAs versus fewer rural women (22.7%, 37.5%, and 78%) in 1998–1999, 2005–2006, and 2015–2016, respectively.

The PNC reported by women in the higher education group during NFHS 3 was more than three times in comparison with illiterate women, whereas it increased by a quarter in the same group during NFHS 4. The percentage of women in younger age groups (15–19 years) had received better PNC than older women. Also, a higher percentage of women belonging to birth order 1, richest quintile, and those living in urban areas reported better PNC.

### Determinants of four or more ANC visits, skilled birth attendance, and PNC

Tables 2, 3, and 4 show the effects of various demographic and socioeconomic indicators on the utilization of ANC, skilled birth attendance, and PNC in rural India. There was significant increase in the odds of using prenatal care, skilled birth attendance, and PNC in 2005–2006 and 2015–2016 as compared with 1998–1999. A strong association between education and utilization of maternal health care services was visible. The odds of using ANC, skilled birth attendance, and PNC were higher for women with primary education than for women with no education, whereas the effects of higher education on use of maternal health services were even stronger. Mother's age was negatively associated with making of four of more ANC visits, using skilled birth attendance and PNC. Interestingly, women with parity of two or more did not significantly differ from women with first parity in the use of these services. Health card emerged as an important predictor of health care utilization. Partner's education had a positive impact on maternal health care services use, although the effects were markedly less as compared with mother's own education. Religion and caste showed a significant bearing on utilization behaviors. Muslim and other women were less likely to use all the three services. Surprisingly, other backward class women were less likely to make four of more prenatal visits as compared with scheduled caste women, but in case of skilled birth attendance and PNC, other backward class and upper caste women showed better utilization than scheduled caste women. Women's autonomy is an important predictor of health care use. Freedom to go to health center and holding bank account had a strong positive impact on the utilization of all the three services. Women who used mass media had better odds of utilization of ANC, skilled birth attendance, and PNC than those who did not watch television and read newspaper.

**Table 2.** Determinants of Four of More Antenatal Care Visits in Rural India

	OR	SE	Z	p* > |*Z|	95% CI
Year	1998–Ref.				
2005	1.97	0.07	19.18	0.00	1.83–2.11
2015	2.15	0.08	20.53	0.00	2.00–2.31
Women's education	Illiterate–Ref.				
Primary	1.43	0.08	6.13	0.00	1.28–1.60
Secondary	2.03	0.13	10.89	0.00	1.79–2.31
Higher	7.60	1.86	8.27	0.00	4.70–12.29
Women's age (in years)	15–19 to Ref.				
20–29	0.98	0.08	−0.19	0.85	0.84–1.15
30–39	0.78	0.07	−2.91	0.00	0.66–0.92
40–49	0.55	0.07	−5.00	0.00	0.43–0.69
Parity	1–Ref.				
2–4	1.02	0.05	0.44	0.66	0.92–1.13
5+	0.99	0.06	−0.20	0.84	0.87–1.12
Health card	No–Ref.				
Yes	2.98	0.12	27.39	0.00	2.76–3.23
Partner's education	Illiterate–Ref.				
Primary	1.25	0.07	3.98	0.00	1.12–1.40
Secondary	1.31	0.06	5.52	0.00	1.19–1.45
Higher	1.32	0.13	2.84	0.00	1.09–1.59
Religion	Hindu–Ref.				
Muslim	0.78	0.05	−4.37	0.00	0.69–0.87
Others	0.45	0.03	−12.15	0.00	0.39–0.51
Caste	SCs–Ref.				
Scheduled tribes	0.89	0.06	−1.63	0.10	0.78–1.02
Other backward class	0.89	0.05	−2.09	0.04	0.80–0.99
Upper caste	1.02	0.07	0.29	0.77	0.90–1.16
Autonomy	No autonomy–Ref.				
Go to health center	1.28	0.04	7.52	0.00	1.20–1.37
Bank account	1.37	0.12	3.61	0.00	1.15–1.62
Watching TV	1.57	0.07	9.94	0.00	1.43–1.71
Reading newspaper	1.57	0.07	10.67	0.00	1.44–1.71
Village level factors	No facility–Ref.				
Drainage	1.40	0.06	8.56	0.00	1.30–1.51
Electricity	2.28	0.11	17.31	0.00	2.08–2.51
Clean water	1.39	0.04	10.81	0.00	1.31–1.48
Transport	1.18	0.08	2.64	0.01	1.04–1.34
Standard of living	Poorest–Ref.				
Poorer	1.23	0.10	2.65	0.01	1.06–1.44
Middle	1.22	0.09	2.76	0.01	1.06–1.41
Richer	1.19	0.08	2.50	0.01	1.04–1.36
Richest	1.14	0.08	1.96	0.05	1.00–1.30
LR chi2(30)	9,411.24				
Pseudo R2	0.2907				

*Source:* Based on three rounds of NFHS pooled data.

CI, confidence interval; LR chi2, likelihood ratio chi-square test; OR, odds ratio; R2, coefficient of determination; Ref., refers to reference categories; SCs, scheduled castes; SE, standard error.

**Table 3.** Determinants of Skilled Birth Attendance in Rural India

	OR	SE	Z	p* > |*Z|	95% CI
Year	1998–Ref.				
2005	2.06	0.08	19.86	0.00	1.92–2.22
2015	2.74	0.10	28.62	0.00	2.56–2.94
Women's education	Illiterate–Ref.				
Primary	1.51	0.08	8.14	0.00	1.37–1.67
Secondary	2.42	0.12	17.86	0.00	2.19–2.66
Higher	7.27	0.79	18.33	0.00	5.88–8.99
Women's age (in years)	15–19 to Ref.				
20–29	0.84	0.06	−2.52	0.01	0.73–0.96
30–39	0.81	0.06	−2.82	0.01	0.70–0.94
40–49	0.65	0.08	−3.37	0.00	0.50–0.83
Parity	1–Ref.				
2–4	1.07	0.05	1.51	0.13	0.98–1.16
5+	0.98	0.05	−0.40	0.69	0.88–1.09
Health card	No–Ref.				
Yes	2.14	0.08	20.55	0.00	1.99–2.30
Partner's education	Illiterate–Ref.				
Primary	1.23	0.07	3.65	0.00	1.10–1.38
Secondary	1.22	0.06	4.08	0.00	1.11–1.34
Higher	1.41	0.10	4.84	0.00	1.23–1.62
Religion	Hindu–Ref.				
Muslim	0.85	0.04	−3.30	0.00	0.77–0.94
Others	0.86	0.05	−2.44	0.02	0.77–0.97
Caste	SCs–Ref.				
Scheduled tribes	0.54	0.04	−9.12	0.00	0.48–0.62
Other backward class	1.15	0.05	2.89	0.00	1.04–1.26
Upper caste	1.30	0.06	5.30	0.00	1.18–1.43
Autonomy	No autonomy–Ref.				
Go to health center	1.10	0.04	2.88	0.00	1.03–1.18
Bank account	1.44	0.08	6.80	0.00	1.30–1.60
Watching TV	1.35	0.06	7.28	0.00	1.25–1.46
Reading newspaper	1.54	0.07	10.00	0.00	1.41–1.67
Village level factors	No facility–Ref.				
Drainage	1.43	0.06	9.10	0.00	1.33–1.55
Electricity	1.95	0.09	14.45	0.00	1.78–2.14
Clean water	1.10	0.05	2.42	0.02	1.02–1.20
Transport	1.18	0.07	2.94	0.00	1.06–1.31
Standard of living	Poorest–Ref.				
Poorer	1.98	0.03	46.55	0.00	1.93–2.04
Middle	3.01	0.05	67.11	0.00	2.92–3.11
Richer	3.92	0.07	75.59	0.00	3.79–4.06
Richest	4.33	0.08	77.85	0.00	4.17–4.49
LR chi2(30)	9,411.24				
Pseudo R2	0.2907				

*Source:* Based on three rounds of NFHS pooled data.

**Table 4.** Determinants of Postnatal Care in Rural India

	OR	SE	Z	p* > |*Z|	95% CI
Year	1998–Ref.				
2005	2.46	0.09	25.26	0.00	2.29–2.64
2015	2.61	0.09	28.65	0.00	2.45–2.79
Women's education	Illiterate–Ref.				
Primary	1.53	0.08	8.31	0.00	1.38–1.69
Secondary	2.17	0.11	15.66	0.00	1.97–2.39
Higher	4.11	0.38	15.18	0.00	3.42–4.93
Women's age (in years)	15–19 to Ref.				
20–29	1.10	0.08	1.32	0.19	0.96–1.26
30–39	0.99	0.08	−0.08	0.94	0.86–1.15
40–49	0.74	0.10	−2.32	0.02	0.57–0.95
Parity	1–Ref.				
2–4	1.09	0.05	2.03	0.04	1.00–1.19
5+	0.97	0.05	−0.58	0.56	0.87–1.08
Health card	No–Ref.				
Yes	2.43	0.09	23.85	0.00	2.26–2.61
Partner's education	Illiterate–Ref.				
Primary	1.12	0.06	2.00	0.05	1.00–1.26
Secondary	1.12	0.06	2.37	0.02	1.02–1.24
Higher	1.26	0.09	3.38	0.00	1.10–1.45
Religion	Hindu–Ref.				
Muslim	0.92	0.05	−1.77	0.08	0.83–1.01
Others	0.81	0.05	−3.56	0.00	0.73–0.91
Caste	SCs–Ref.				
Scheduled tribes	0.72	0.05	−5.02	0.00	0.63–0.82
Other backward class	1.19	0.06	3.75	0.00	1.09–1.31
Upper caste	1.26	0.06	4.69	0.00	1.14–1.39
Women's autonomy	No autonomy–Ref.				
Go to health center	1.28	0.04	7.52	0.00	1.20–1.37
Bank account	1.54	0.08	8.40	0.00	1.39–1.71
Watching TV	1.43	0.06	8.54	0.00	1.32–1.55
Reading newspaper	1.57	0.07	10.67	0.00	1.44–1.71
Village level factors	No facility–Ref.				
Drainage	1.40	0.06	8.56	0.00	1.30–1.51
Electricity	2.28	0.11	17.31	0.00	2.08–2.51
Clean water	1.21	0.05	4.45	0.00	1.11–1.32
Transport	1.03	0.02	2.11	0.04	1.00–1.06
Standard of living	Poorest–Ref.				
Poorer	1.15	0.07	2.55	0.01	1.03–1.29
Middle	1.22	0.06	3.77	0.00	1.10–1.35
Richer	1.18	0.06	3.42	0.00	1.07–1.29
Richest	1.11	0.05	2.40	0.02	1.02–1.21
LR chi2(30)	8,433.65				
Pseudo R2	0.2645				

*Source:* Based on three rounds of NFHS pooled data.

The results with respect to village level factors revealed that improvement in infrastructure was related to better utilization of maternal health care services. Provision of drainage facility, village electrification, availability of clean water, and transport facility corroborated with greater odds of having taken adequate ANC visits, skilled birth attendance, and PNC. The economic indicators (standard of living) were stratified using wealth quintiles and a significant association of income was observed with ANC, skilled birth attendance, and PNC utilization.

## Discussion and Conclusion

This study examined the strength of association of the use of maternal health care services namely four or more ANC visits, delivery assisted by skilled birth attendance, and PNC, with selected sociodemographic and economic predictors at grass root level in rural India. The findings of the study revealed an upward trend in four or more ANC visits, deliveries assisted by SBAs, and receipt of PNC from 1998–1999 to 2015–2016. Female literacy was found to have significant association with four or more ANC visits, skilled birth attendance, and PNC. This clearly indicates that education directly translates into better health-related behavior, increased comprehension, and compliance of medical advice, thereby improving the overall well-being of a family. Better utilization of maternal health care services naturally follows modest education and is supported by other studies.^31,32^ Partner's education also plays a significant role in supporting a woman's access to ANC, skilled birth attendance, and PNC.^33^ Moreover, women with better economic conditions by virtue of higher education may influence maternal health by controlling the extent to which they can access and afford health care services.^34^ Educated women have higher income through better occupation^35^ and may “meet” educated and higher income partner.^36^ Women with gainful occupation may also postpone childbearing.^37^ Education increases women's emoluments, thereby enhancing their ability to provide financial support to their families and thus participate in household decision-making activities,^38^ including decisions on fiscal spending on their own as well as child health care.^16^ Moreover, educated women are well informed about modern health care and have better ability to communicate with health care providers.^39^ However, maternal age showed an inverse association with utilization of maternal health care services.^40^

The finding that possession of heath cards was associated with a higher utilization of prenatal care, skilled birth attendance, and PNC may be explained by the fact that the ANM who fills up the health card offers basic antenatal services and provides one-on-one information and advice regarding maternal and child health and family planning, thereby acting as a catalyst of increased utilization of maternal health care services. It is expected that an ANM visits every household in a village, identifies the pregnant women, enrolls them in the antenatal register, provides prenatal care, identifies any complications, and also provides postnatal services.^41^ Although the quality of services offered by an ANM at village level may be compromised,^42^ it most certainly reduces monetary and opportunity cost that by default leads to an increase in the use of maternal health care services.^43^

The results of the study are in congruence with the notion that female autonomy increased the probability of receiving maternal health care though control of individual and social factors. Autonomy in terms of financial independence and decision making (visiting to health facility center) was significantly associated with use of maternal health care, which is in tune with other findings.^44–46^ Media exposure, that is, watching television and reading newspaper once a week boosted use of maternal health services, which is in agreement with other studies.^47,48^ Media exposure accentuates awareness about health care needs, sensitizes general population about the available health services, and facilitates acceptance of modern medical services, thus enabling better utilization of maternal health care services particularly in rural areas.

Village level factors were also strongly associated with the use of ANC services, skilled birth attendance, and PNC. Satisfactory economic development at village level, reflected by household amenities (such as drainage facility, electrification, and clean water), basic public utilities such as transport facility, and greater purchasing power of goods and services (standard of living was measured by the wealth quintile), leads to greater use of maternal health care services.^49^ It is known that investments in infrastructure at a village level, especially drainage, electrification, water, road, and transport, increase the probability of maternal health care utilization.^50^ Our analysis also suggested that physical access to health care through transportation increases the utilization of maternal health care services, which is in line with other studies.^51^ These village level factors independently predict the use of ANC, SBA, and PNC, thereby indicating that drainage facilities, village electrification, availability of clean water, transport facilities, and standard of living are positively associated with the use of all three maternal health care services.

### Policy suggestions

Thus, the findings of this study sketch a broad blueprint for mapping out a plan of action to improve maternal health care services utilization. This blueprint includes (1) relentless focus on timely ANC with four or more visits as only 51% of women used prenatal care during pregnancy, (2) increase the delivery of postpartum care, because 44.65% and 14.88% of women reported severe bleeding and high fever, respectively, after delivery, (3) reduce the socioeconomic inequalities in pregnancy care, because findings reveal that women with lower income level and those belonging to scheduled tribes and scheduled castes are less likely to use maternal health care services, (4) improve women's education level, because women with higher education, use 7.6 times more ANC, are 7.25 times more likely to deliver their child in the presence of an SBA and are 4.11 times more likely to use PNC, thus reiterating the fact that female literacy is a bedrock for a healthy nation, (5) ensure freedom of mobility, financial freedom, and freedom to use mass media, as these factors empower women, thereby increasing the probability of maternal health care use, and (6) the village level factors were significantly associated with the use of maternal health care, therefore, it is necessary to design a comprehensive model to improve public health infrastructure within villages.

However, these strategies can only be fructuous when advocacy, political commitment, and adequate funding are coupled with underpinning values of community participation, intersectoral coordination, appropriate technology, and equitable distribution so as to ensure provision of health care services that are affordable, accessible, acceptable, and accountable, thereby creating an enabling environment to reach highest attainable standard of health and well-being for every individual. Thus, with only a decade till the SDGs are due, a lot needs to be done to help reduce maternal mortality and enhance universal coverage of health services across the nation.^3^

### Limitations

This study used data from three rounds of NFHS collected during 1998–1999, 2005–2006, and 2015–2016, which is based on self-reported information of individuals, is subject to recall bias, and is not validated from different sources. This study reviewed the factors affecting maternal health care services utilization by examining India as a whole rather than any specific community, district, or state. So, this study does not provide micro-level analyses that may be provided from community-level studies. The analytical approach is not comprehensive because all the predictors could not be included in the estimations. However, the study was able to include predictors such as possession of health card and basic amenities such as drainage facility at local level, electrification, and availability of clean water that have not been included in previous studies. There may also be other biases due to unmeasured factors. However, it does furnish a useful population level analysis of the utilization of maternal health care services and its determinants with emphasis on rural India. These findings can support decision makers in planning health-related schemes at national level and formulate hypotheses for future research.

## Abbreviations Used

ANC: antenatal care
ANM: auxiliary nurse midwife
CI: confidence interval
MDGs: millennium development goals
MMR: maternal mortality ratio
NFHS: National Family Health Survey
NRHM: National Rural Health Mission
OR: odds ratio
PNC: postnatal care
SBA: skilled birth attendant
SCs: scheduled castes
SDGs: sustainable developmental goals
SE: standard error
WHO: World Health Organization
